# Supplementary material for: Serum Endocan Is a Risk Factor for Aortic Stiffness in Patients Undergoing Maintenance Hemodialysis
Source: Medicina (Kaunas). 2024 Jun 14;60(6):984. doi: 10.3390/medicina60060984 (PMC11205908; doi:10.3390/medicina60060984)
Supplement: Supplementary file 1 [file medicina-60-00984-s001.zip › medicina-3026457-supplementary.pdf]

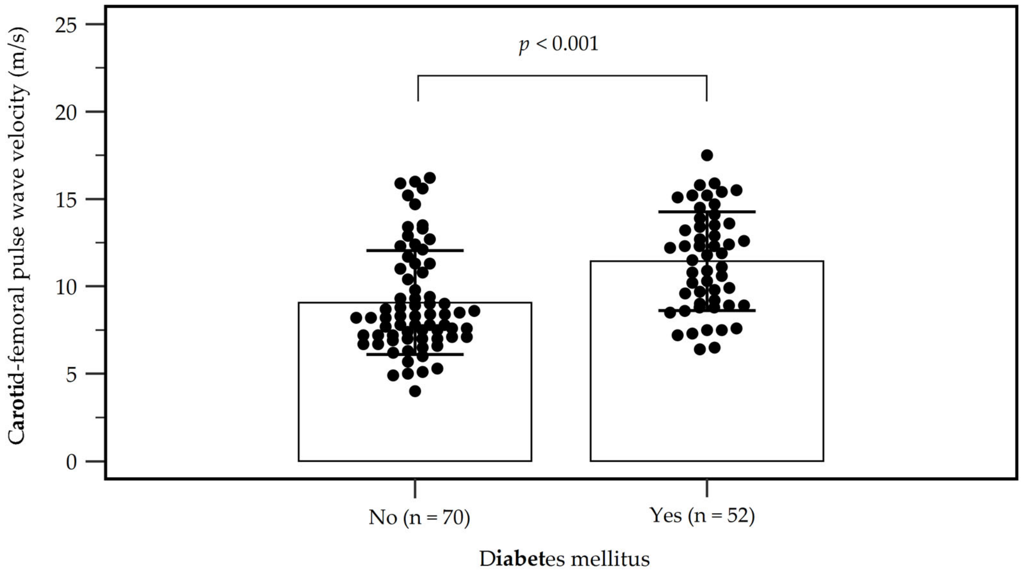

Supplementary Figure S1. Relationships between carotid-femoral pulse wave velocity (cfPWV) and 122 hemodialysis patients with or without diabetes mellitus. The cfPWV among HD patients with or without DM by mean ± standard deviation and was compared between the two groups using a two-tailed independent Student's t-test (p < 0.001).

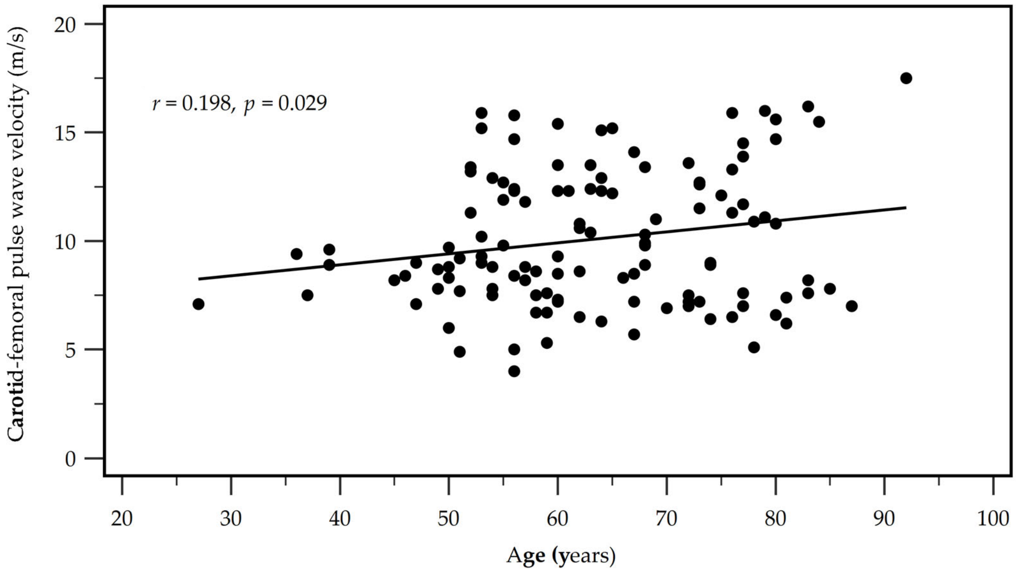

(a)

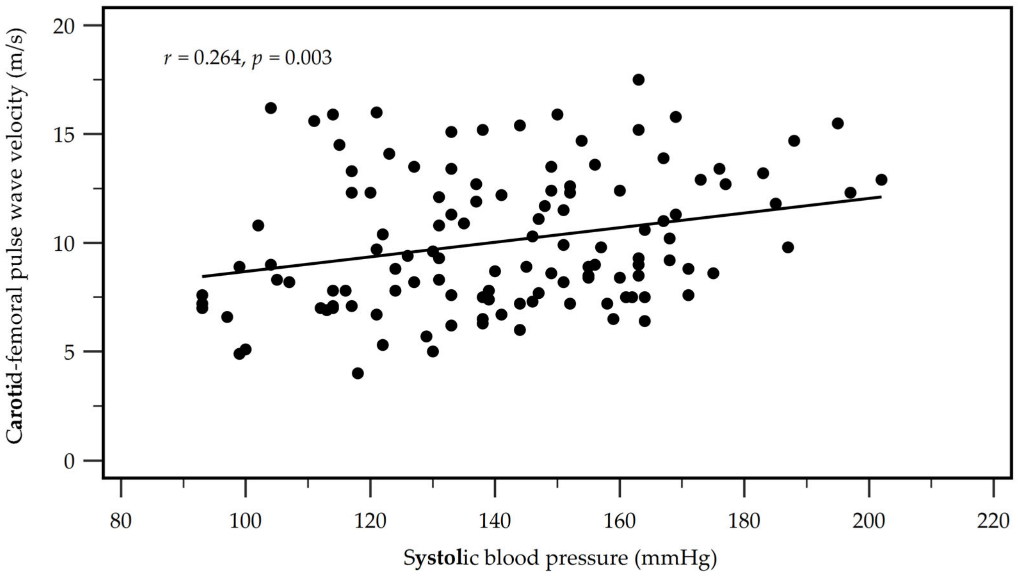

(b)

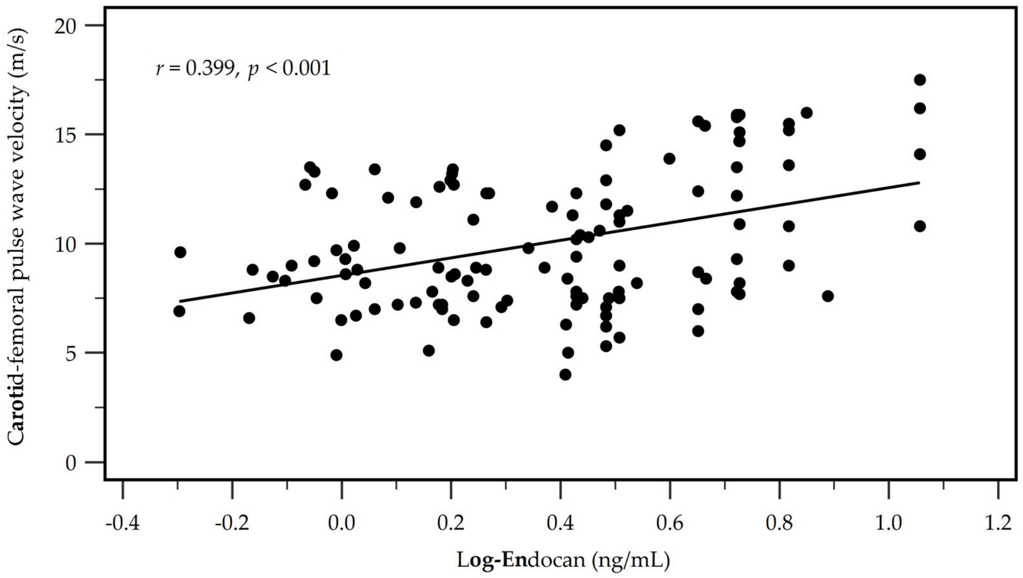

(c)

Supplementary Figure S2. Relationships between carotid-femoral pulse wave velocity (cfPWV) and (a) age, (b) systolic blood pressure, and (c) log-transformed endocan (log-endocan) among 122 hemodialysis patients. Two-dimensional scatter plots of cfPWV values with age, systolic blood pressure, and serum log-endocan level among patients undergoing HD are presented in a to c, respectively.
